# Supplementary material for: Patterns of Intron Gain and Loss in Fungi
Source: PLoS Biol. 2004 Nov 30;2(12):e422. doi: 10.1371/journal.pbio.0020422 (PMC532390; doi:10.1371/journal.pbio.0020422)
Supplement: Table S1 — Also available at http://genes.mit.edu/NielsenEtAl/. (4.3 MB ZIP). [file pbio.0020422.st001.zip › NielsenEtAl/html/48.html]

AN0352.1.NCU00743.1.MG00063.1.FG09704.1


```
 CLUSTAL W (1.82) Multiple Sequence Alignments - Introns Inserted


Sequence 1: NCU00743.1	1530 aa
Sequence 2: MG00063.1	1588 aa
Sequence 3: FG09704.1	1553 aa
Sequence 4: AN0352.1	2376 aa
Alignment Length: 2471 aa
Number Identitical Residues: 756 aa
Alignment Score (without introns) 39869


MG00063.1 	-MIPRTMISNEVYYLPLNDDGSPQVQGEYIYLAPKSNEPFTIRFAIEGTSSICRQGSLWV
NCU00743.1	-MIPRTMVSNEVYLLPLKDDGSPDVPGEYIYIAPKSKDPVTIRFAIEGTSSICSHGSLWV
FG09704.1 	MSPPHTMTSNEVYLLPLNDDGSPQVQGEYIYLAPRSQEPITVRFAIEGTSSICRHGSLWV
AN0352.1  	------MSNETFYLLPLNDDGSPDVPGGFIYLPPPDHPKYSLRFLIEGSSSICRDGTLWI
          	      * .: .* ***:*****:* * :**:.* .:   ::** ***:**** .*:**:

MG00063.1 	NIPEPGVEFRRDQFREFK2LVPDFNRTIEISIPVHLPGAYAFYTTYAKLPELEQTAG-AD
NCU00743.1	NIPEHGEQFQRNKFREFK2LVPDFNRTLEISIPIYEAGAYAFYTTYAELPDLASNLVNAD
FG09704.1 	NIPDEGAEFRRDHFREFK2LTPDFNRTLEISIPIHQPGAYAFYTTYAELPELKKELK--N
AN0352.1  	NIPEIGKSFDRQSFRPFK2LYPDFSRNIQIDVPVTCPGSFAYYVTYSPLPEFTITSS---
          	***: * .* *: ** ** * ***.*.::*.:*:  .*::*:*.**: **::        

MG00063.1 	SSPSSTDKNKTQLYYIDVAPRLQLDGRPLPLPALSIFSVISKFMGKYPNDWERHLRGISD
NCU00743.1	GTVTKATQKKTPLYYIDVAPRLSLDGQPLPLPALSIFSIISKFMGKYPTDWERHLRGISD
FG09704.1 	SSEQKENLKKTPLYYIDVAPRLKLDGRPLPLPALSIFSVISKFMGKYPNDWERHLHGISD
AN0352.1  	---APVKPARTPTHYIDVSPRLSLAGEGIPLNALSIFSVNSKFMGKYPEDWNQYLNSISR
          	         :*  :****:***.* *. :** ******: ******** **:::*..** 

MG00063.1 	RGYNMIHFTPLQTRGTSNSPYSLYDQLSWDPECFPEGEKDIKKLVDSLEKNHSLLSLTDI
NCU00743.1	RGYNMIHFTPLQVRGASNSPYSLYDQLGWDPACFPAGEPDVQKMVESLEKNHSLLSLTDI
FG09704.1 	RGYNMIHFTPLQIRGISNSPYSLYDQLGWDPACFPAGEDDVQKMVESLERNHSLLSLTDI
AN0352.1  	RKYNMVHFTPLMKRGASNSPYSIFDQLQFDDIAFPNGENDVQKLIKDMEEKHSLLSLADV
          	* ***:*****  ** ******::*** :*  .** ** *::*::..:*.:******:*:

MG00063.1 	VLNHTADNSEWLLEHPEAGYNLTTAPWLELPFLIDTKLLELGFNLAKLGLPTEVKSEADV
NCU00743.1	VLNHTAHNSEWLLEHPEAGYNLTTAPWLESAYLLDTKLLELGTRLEELGLPTELKDVDDL
FG09704.1 	VLNHTANNTKWLEEHPEAGYNLLTAPWLESAYQLDTSLLELSDNLAKLGLPTDVKSTDDL
AN0352.1  	VWNHTANNSKWLEEHPEAGYSVETAPWLEAALELDTALLKFSHELQKRGLPTDFKTVDDL
          	* ****.*::** *******.: ****** .  :** **::. .* : ****:.*   *:

MG00063.1 	LAIMDAVKKEVISAIRMWEYYVVDVESNAEVAAEAWAAGKGSFPDGSLGTEG-PAGLKSA
NCU00743.1	VKIMDAIKKEVIAEIRLWEYYTLDVERDADAAVKSWAANDIDFPQASVGAGG-IDSLHSA
FG09704.1 	MLIMDAIKTEVLAKIRLWEYYALDVDRDADEAIKAFSKGSKYSSDDTADFEKKLEKAKSG
AN0352.1  	TLVMNEVKNHVIEDLCLWEFYVLDVKSNARRVIDEWKVSTNSDIERGRWAQLDLGAYKAW
          	  :*: :*..*:  : :**:*.:**. :*  . . :  .     :       .    :: 

MG00063.1 	SLSERAQFVIKYGLSGTDRMGERFRRKVVPEVAAGVLDALLGRPEGHQGPDSGAA~RQKM
NCU00743.1	TPKEQADFLIQHGLQNMDYL----------------------------------A1RTKI
FG09704.1 	SVKEQVDFFREVGLAGTDRMGERFRKRVKPDVAASFLAG---------SSDESAA~RAKI
AN0352.1  	SLEDQASLIRQKGVPTAGQILGRYSREIDTQLGVAIATALFG-NFDATSSDLTAV~ENTL
          	: .::..:. : *:   . :         .. . .   .  .   .  ...  :. . .:

MG00063.1 	IEILQAVNVPFYEEYDKDSAEILEQTFNRIKYVRLDEHGPKLGPINEANPLVETYFTRLP
NCU00743.1	VEILEIVNVPFYKEYDDEVAEILQQLFNRIKYVRLDDHGPKLGPINAENPLIETYFTRLP
FG09704.1 	VEILNILNVDFYKEYDAEVDDILQQIFNRIKYVRLDEHGPKLGEINKENPLIEPYFTRLP
AN0352.1  	RKLLDVVNVPFYKEYNSDVTEIMSQLFNRIKYLRIDDHGPKLGPVTDQNPLIESYFTRLP
          	 ::*: :** **:**: :  :*:.* ******:*:*:****** :.  ***:*.******

MG00063.1 	RNEKTAKHKKEDLVLVNNGWVWGGNALVDNAGPDSRVYLRREVIVWGDCVKLRYGAGPED
NCU00743.1	VNEKTKKHKKEDLVLANNGWVWGGNALVDNAGPDSRVYLRREVIVWGDCTKLRYGSGPED
FG09704.1 	KNDTTSKLKPEEMALVNNGWVWGGNALVDNAGPDSRVYLRREVIVWGDCVKLRYGSGPKD
AN0352.1  	LNEITKKHSPKALALANNGWIWNADAMRDNAGPNSRAYLRREVIVWGDCVKLRYGDGPED
          	 *: * * . : :.*.****:*..:*: *****:**.************.***** **:*

MG00063.1 	SPYLWDRMTKYSRMLAKYFAGFRIDNAHSTPIHVAEHILDEARRVRPDLYVCGELFTGSE
NCU00743.1	SPWLWEHMTKYARMLAKYFAGFRIDNCHSTPLHVAEHILDEARRVRPDLYVVAELFTGSE
FG09704.1 	NPWLWEHMTKYARTLAKYFAGLRIDNCHSTPIHVAEHILDEARRVRPDLYVVAELFTGSE
AN0352.1  	NPFLWDFMARYTRLMAKYFSGFRIDNCHSTPLVVAEYLIDEARKIRPDLAIFAELFTGSE
          	.*:**: *::*:* :****:*:****.****: ***:::****::**** : .*******

MG00063.1 	EMDYVFVKRLGLSSLIREAMQAWSTGELSRLVHRHGGRPIGSFEVDEVSRGDASPTSPHP
NCU00743.1	EMDYVFVKRLGISALIREAMQAWSTGELSRLVHRHGGRPIGSFEVDEVSSNEGRSSS---
FG09704.1 	EMDYVFVKRLGLSSLIREAMQAWSTAELSRLVHRHGGRPIGSFEVDEVSKSDVRTPSSSP
AN0352.1  	EADYIFVKRLGINALIREAMQAWSAGELSRLVHRHGGRPIGSFELDLPSPGLDLSKS---
          	* **:******:.:**********:.******************:*  * .   . *   

MG00063.1 	QSPQVNGGTTDEMWSSREIIRRIKPSPVQALFMDCTHDNEVPAQKRDARDTLPNAALVSM
NCU00743.1	-----ISGTNGDGVYTREVIRRIRPVPVQALFMDCTHDNEVPAQKRDARDTLPNAALVAM
FG09704.1 	TRLK-NGDTNGNGPRSREIIRAIKPSPVHALFMDCTHDNETPAQKRDARDTLPNAALVCM
AN0352.1  	----------QVSGSEREIIRSIRPIPVPALFMDCTHDNEMPAQKRTATDTLPNAALVAM
          	                **:** *:* ** *********** ***** * *********.*

MG00063.1 	CASATGSVMGYDEIYPKLVDLVNETRLYTSESS--AREVKVGSGKGGIGGLKKLMNQIHT
NCU00743.1	CASATGSVMGYDEIYPKLVDLVGETRLYTSEAS--KAPVKTGEGKDGIAGVKKLLNQIHT
FG09704.1 	CSSATGSVMGYDEIYPKLVDLVNETRLYTSASSG-SKPIKIGGGEDGIGGIKKLLNQIHT
AN0352.1  	CDSAIGSVMGYDEVYPKIVDLVHETRLYSFFDLPESPTLDELVNLKGISGLKRLLNELHT
          	* ** ********:***:**** *****:     .   :.   .  **.*:*:*:*::**

MG00063.1 	LMGKDGYDETHIHHEDQYITVHRVHPQSRKGYFLIAHTAYPGYGNGNGAFNPVHLTGTKV
NCU00743.1	LMGMDGYDETHIHHEDEYVTVHRVHQESRKGYFLIAHTAFPGYGNGNGAFNPVHLTGTKA
FG09704.1 	LMGKDGYDETHIHHEDQYITVHRVHPESRKGYFLIAHTAFPGYGNGNGDFSPVHLTGTKA
AN0352.1  	MMGQMGYDETFIHHDGEYITVHRVHPQTRKGIFLIAHTAFPGQNSG-ALLAPIHLAATRA
          	:**  *****.***:.:*:****** ::*** *******:** ..* . : *:**:.*:.

MG00063.1 	RHLGSWTLEVDDSEEARKNVLEDKKHLRGLPSRVIDLPGIRMEVKGSDTVITVRDRFPPG
NCU00743.1	RHLGSWMLEVDASKEAVEEVLSDKKHLRGLPSRLVGLPGVRMEVKGQDTIITVREKFPPG
FG09704.1 	RHLGSWMLEVDASEEATRQALSDKKYLRGLPSRVKDVPGIRMEVNGDDTTITVRDRFPPG
AN0352.1  	KPIGSWLLKVQELSDDKERALSDKVYLQGLPSEVLKIEGARIEETDRETIISVLDPFIPG
          	: :*** *:*:  .:  ...*.** :*:****.:  : * *:* .. :* *:* : * **

MG00063.1 	SIALFETYIPAAEHSAGLDT-YVTSQAQDAFAGLNLIDMNFMMYRCEAEERDWSGGSDGV
NCU00743.1	SIALFETWIPAAEHSSGLDN-FVTSGAKAAMDELDLVDLNFLLYKCEPEERDASEGQDGT
FG09704.1 	SIALFETWIPAAEHSSGLDN-YVTSGAKAAWSELSLSDLNFLMYRCEAEERDESDGRDGV
AN0352.1  	SIALFETSIPSLEHADELSNDAITDGAVEAFSELSLIDLNFVLYRCDAEERDSSGGQDGV
          	******* **: **:  *... :*. *  *   *.* *:**::*:*:.**** * * **.

MG00063.1 	YGIPGHGNLVYAGLQGWWSLLEGIIRDNNLAHPLCQNLREGQWALDFIIGRLERASKTPE
NCU00743.1	YDIPGHGKIVYAGLQGWWSILKDVIKDNNLAHPLCQHLRDGQWALDYIVGRLERASKKDD
FG09704.1 	YDIPGHGKLVYAGLQGWWSILKNIIKENNLAHPLCQNLRDGEWALDFILARLQRISATPG
AN0352.1  	YDIPNHGPLVYAGLQGWWSILEGIIRYNELGHPLCDHLRQGQWALDYIVNRLMKATKNPQ
          	*.**.** :**********:*:.:*: *:*.****::**:*:****:*: ** : : .  

MG00063.1 	FSRLEKPTRWLKDRFDAIRGIPSFLLPRYFALVMRTAYMACFERSVSLMNENVAKGQWFL
NCU00743.1	FRRLAKPAQWLKERFDAIRPIPSFLLPRYFGLVLRTAYNAAFERGISLMNNNVIKGQWFL
FG09704.1 	NQAVAEPLKWLQERFEAIRKIPSFLLPRYFGLVLRTAYMASWERSLQLMNASVRDGQWFL
AN0352.1  	YAALRRPAEWLEAKFQIVRGLPSFLLPRYFAIIVQTAYVAAWNRGIQLLGDDVRVGQAFT
          	   : .* .**: :*: :* :*********.::::*** *.::*.:.*:. .*  ** * 

MG00063.1 	KSLAMVSTQETGIVKSASLYPDHLVPSLAAGLPHFAVDWARCWGRDIFISLRGLFLGTGR
NCU00743.1	QSLAMVSVQMTGLVKSASLYPNRLVPSLAAGLPHFAVEWARCWGRDVFISLRGLYLGTGR
FG09704.1 	QSLAMVSVQQVGYVKSASLWPNKLVPSLAAGLPHFAVEWARCWGRDVFIALRGLLLGTGR
AN0352.1  	QQLAMVSVQQTGFVNSASLWPKKSVPSLAA------------------------------
          	:.*****.* .* *:****:*.: ******                              

MG00063.1 	FDEAREHILAFASVLKHGMIPNLLG~AGRTPRYNARDSIWFFLQCIQDYTRLVPNGLSLL
NCU00743.1	YAEAREHIHAFASVLKHGMIPNLLG~SGNNPRYNSRDSVWFFLQCIQDYTRLVPDGLSIL
FG09704.1 	FDDAKEHILAFASVLKHGMIPNLLS~SGDAPRYNSRDSIWFFLQCIQDYTRFAPEGLDIL
AN0352.1  	------------------------G1SGKLPRYNSRDSIWFLLQAIQDYTKMAPDGLELL
          	                        . :*  ****:***:**:**.*****::.*:**.:*

MG00063.1 	DEKVKRRFLPYDDTYFDAEDSRAYSTQSTIRDIIQEALQRHASGMSFREANAGPSLDMQM
NCU00743.1	DDKVKRRFLPYDDTYFDVDDERAYSKESTIAEIIQEVFQRHAEGMKYREANAGPNLDMQM
FG09704.1 	KVKVKRRFLPYDDTWFPTDDERAYSKESTIEEVIQEALQRHATGMKYREANAGPQIDSQM
AN0352.1  	QDKTPRRFLPYDDTWFPFDDPKAYSQTSTIVDIIQEVFQRHAQGISFREYNAGPELDVQM
          	. *. *********:*  :* :***  *** ::***.:**** *:.:** ****.:* **

MG00063.1 	SDKGFDLEVKVDWDTGFVLGGNQFNCGTWMDKMGESEKAGSKGVPGTPRDGAAVEITGLM
NCU00743.1	SDAGFNQEIKVDWSNGFIFGGNQANCGTWMDKMGESERAGSKGVPGTPRDGAAVEITGML
FG09704.1 	KDEGFNQDIHVDWNNGIIFGGNQDNCGTWMDKMGESERAGSKGVPGTPRDGAAIEITGML
AN0352.1  	KSEGFNIDIRVNWETGLIFGGSQHNCGTWQDKMGESEKAGNKGVPGTPRDGAAIEITGLL
          	.. **: :::*:*..*:::**.* ***** *******:**.************:****::

MG00063.1 	YSTVKWVAKLHSSGEFPYSGVSKAAVGPDQDPKSVNVDVSYADWAELVKSNFERCYFVPL
NCU00743.1	YSNLKWAASLNEAGKFKHSSVRKADG----------TEITLKDWAALIKDNFERCYFVPI
FG09704.1 	YSTLDWLAGLHGKGKYAYAGVEKSDG----------SSISLADWASLLKANFERCYYVPI
AN0352.1  	YSALKWVTELNKQGLFPYDKVDIGNG----------KSITFEGWAAKVKTNFERCYYIPL
          	** :.* : *:  * : :  *  .             .::  .**  :* ******::*:

MG00063.1 	DPSEDSKYDVNPSVINRRGIYKDLYRSGKEYEDYQLRPNFPIAMTVAPDLFDEAHAMHAL
NCU00743.1	SPDEDSKYDVNPAIINRRGIYKDLYRSGKEYEDYQLRCNFPIAMTCAADLFVPEHAMHAL
FG09704.1 	SAEDDSKYDVNTPIVNRRGIYKDLYKSGKEYEDYQLRPNFAIAMTTAPALFDPDHAMHAL
AN0352.1  	DPNDDDQYDIDSGIVNRRGIYKDLYRSGKPYEDYQLRSNFPIAMVVSPELFDPSKALGAL
          	...:*.:**::. ::**********:*** ******* **.***. :. **   :*: **

MG00063.1 	CIADQVLRGPQGMATLDPADLNYRPYYVNSEDSTDFATSKGRNYHQGPEWLWPTGFFLRA
NCU00743.1	WVADAALRGPTGMATLDPSDMNYRPYYNNSEDSDDFATSKGRNYHQGPEWLWPTGFFLRA
FG09704.1 	CVADEALRGPQGMATLDPADLNYRPYYVNSEDSDDFATSKGRNYHQGPEWIWPTGFFLRA
AN0352.1  	AIADSVLAGPLGMATLDPSDLNYRPDYNNAEDSTDFATSKGRNYHQGPEWVWLRGYFLRA
          	 :** .* ** *******:*:**** * *:*** ****************:*  *:****

MG00063.1 	LLKFLLKRTHSAEDGESAATEAFQQVTRRLMGCKAMIKESPWAGLQELTQKDGGFCGDS0
NCU00743.1	LLKFDLMR--RGRDDAEGRTEAFQQVTRRLIGCKEMIQRSPWAGLTELTNKNGEECHDS0
FG09704.1 	LLKFDLKR----RTTAEDRTEAFQQVTRRLGGCKKMIQESPWAGLQELTQKNGEYCADS0
AN0352.1  	LLYFDLAR----RKTADERTEAFQQVTRRLAGCKKALRESPWKGLTELTNRNGNHCADS0
          	** * * *    .   .  *********** ***  ::.*** ** ***:::*  * ** 

MG00063.1 	CPTQAWSAS-----------------------CLIDLYMDADE~EQARMQKGVGVNGSSQ
NCU00743.1	SPTQAWSAG-----------------------CLIDLYMDAAE~EQAKLEK----HSLPL
FG09704.1 	SPTQAWSAG-----------------------CLIDLYMDAVE~EQNGA-----------
AN0352.1  	RPSIVKTNCRYSLASKTRSPLQGGLREYHVWYCYRSLALDKPD2CSSTVVRLEKQHSASQ
          	 *: . :    : ::.: :. ...  .     *  .* :*  :  .          . . 

MG00063.1 	KN----------------------------------------------------------
NCU00743.1	R-----------------------------------------------------------
FG09704.1 	------------------------------------------------------------
AN0352.1  	CFNNGANPSTHLRRQEPPLHKAMSLTESVAAMATAITVANHHGTNNGTDDDGPGRKWDDQ
          	  ...:..::    ....  .: : :.: :: ::: : :.  .:...:...... . ...

MG00063.1 	------------------------~-----------------------------------
NCU00743.1	------------------------~-----------------------------------
FG09704.1 	------------------------~-----------------------------------
AN0352.1  	LKGDLYTQLVISLALGITAFLSFC0FLRPKWTELYAARRRQRRAALYLPELPDSFFGWIP
          	 ...  :.   : : . ::  : .    .. :.  ::   .  ::   .. ..:  .  .

MG00063.1 	--------------------~---------------------------------------
NCU00743.1	--------------------~---------------------------------------
FG09704.1 	--------------------~---------------------------------------
AN0352.1  	VLWKITEEQVLQSAGLDAFV0FLSFFRFAIRFTSTVFILAFVVLLPIHYSYTKKLGIPDW
          	   . :...  .::. .:     :    :   :::    :     .   : :.. . .. 

MG00063.1 	------------------------------------------------------------
NCU00743.1	------------------------------------------------------------
FG09704.1 	------------------------------------------------------------
AN0352.1  	DKSIDVGEDGKKKFIDDPPYLWTYVVFTYIFTGLAIFMLFQETKKIIQTRQKYLGSQTST
          	..: . .......  ....   :    :   :. :     ..:..  .: ..  .:.:::

MG00063.1 	------------------------------------------------------------
NCU00743.1	------------------------------------------------------------
FG09704.1 	------------------------------------------------------------
AN0352.1  	TDRTIRLSGIPAEMGSEENIREFIEGLHIGEVESITLCRNWSSLDHLIEERLKVLRNLET
          	:. :   :. .:. .:...  .  ..   .. .: : . . :: .   ..  .   . .:

MG00063.1 	------------------------------------------------------------
NCU00743.1	------------------------------------------------------------
FG09704.1 	------------------------------------------------------------
AN0352.1  	SWVQYLGYKRVRKSGDTLPLRRQPIDSSIFSEDDERMRLLLENGQDDAFDRSRKRPMVRL
          	:  .  . .   .:..: .   .. .::  :....      ......: . : . .    

MG00063.1 	------------------------------------------------------------
NCU00743.1	------------------------------------------------------------
FG09704.1 	------------------------------------------------------------
AN0352.1  	WYGPLKLRYRKVDAIDYYEERLRRLDEEIQSARQKEYPPTELAFVTMKSIAAAQMLVQAI
          	  .. .    . .: .  ..     ... .:: ... ..:. :  : .: :::.   .: 

MG00063.1 	------------------------------------------------------------
NCU00743.1	------------------------------------------------------------
FG09704.1 	------------------------------------------------------------
AN0352.1  	LDPHPMKLLARLAPAPADVIWKNTYLPRARRMFQSWSITVLICFLSVFWSVLLVPVGTLL
          	 .. . .  :  :.:.:.   ..:  . :    .: : :   .  :   :    . .:  

MG00063.1 	------------------------------------------------~-----------
NCU00743.1	------------------------------------------------~-----------
FG09704.1 	------------------------------------------------~-----------
AN0352.1  	KWETLHKVLPQLADALARHPLVKSLVTTGLPTLAFSLLTVAVPYLYNW1LSNHQGMMSRG
          	. .:  .  .. :.: :  .  .:  ::. .: : :  : : .   .   :. ..  : .

MG00063.1 	------------------------------------------------------------
NCU00743.1	------------------------------------------------------------
FG09704.1 	------------------------------------------------------------
AN0352.1  	DIELSVISKNFFFSFFNLFVIFTVIGTATNFYGLWEHLRDSFKDATTIATALANSLENLA
          	. . :  :..   :  .     :  .:::.  .  .   .: ..::: ::: :.: .. :

MG00063.1 	------------------------------------------------------------
NCU00743.1	------------------------------------------------------------
FG09704.1 	------------------------------------------------------------
AN0352.1  	PFYMNVFVLQGLGLFPLKLLEVGSVFLYPINYLMAKTPRDYAELSTTATFSYGYSIPQSI
          	.   .    .. .  . .  . .:    . .   :.:. . :. ::::: : . : ..: 

MG00063.1 	------------------------------------------------------------
NCU00743.1	------------------------------------------------------------
FG09704.1 	------------------------------------------------------------
AN0352.1  	LILVICVIYGVFPASWLICFFGLVYFTIGNFIYKYQLLYAMDHRQHSTGRAWPMICNRVL
          	     .   .  .::   .  .    : ..   . .   : .  . ::. : .  ..   

MG00063.1 	------------------------------------------------------------
NCU00743.1	------------------------------------------------------------
FG09704.1 	------------------------------------------------------------
AN0352.1  	VGLVVFQLAMAGTLGLRKAITLALLIVPLIGATVWFSYFYSQSYEPLTKFIALKSIYRDT
          	 .    . : :.: .  .: : :    .  .::   :   :.: .. :.  : .:   .:

MG00063.1 	------------------------------------------------------------
NCU00743.1	------------------------------------------------------------
FG09704.1 	------------------------------------------------------------
AN0352.1  	PTSGDISPSTTSTFSPPLALDRDAFPIRLGGQVLGLKLKRYVNPSLILPLDSAWLPGRNP
          	.::.. :.::::: :.. : . .: .   ...  . . .   ..:   . .::  .. ..

MG00063.1 	-------------------
NCU00743.1	-------------------
FG09704.1 	-------------------
AN0352.1  	MPELQEDFEYYEDQNHVSV
          	 .. ... .  ....  :
```
